# An updated framework for task shifting and sharing: refining the SHIFT-SHARE based on stakeholder feedback from India

**DOI:** 10.7189/jogh.16.03008

**Published:** 2026-03-20

**Authors:** Shukanto Das, Liz Grant, David Fearon, David Weller

**Affiliations:** 1 University of Edinburgh, Usher Institute, Centre for Global Health, Edinburgh, UK; 2 University of Edinburgh, Usher Institute, Centre for Population Health Sciences, Edinburgh, UK

## Abstract

Shortages of health care providers necessitate task shifting and sharing (TS/S), yet few implementation frameworks guiding these strategies exist. In 2023, we developed the first iteration of the Strategic Healthcare Implementation Framework for Task Shifting, Sharing, and Resource Enhancement (SHIFT-SHARE), a six-stage cyclical framework drawing on change management theories and implementation science. Here we present SHIFT-SHARE 2.0, updated to incorporate stakeholder consultations in India, where we ran eight focus group discussions with 35 stakeholders (November 2023 to September 2024), including health care staff, organisational leaders, academics, and students. Participants reviewed SHIFT-SHARE materials beforehand and gave feedback on its accessibility, terminology, structure, and utility, after which we mapped the framework’s strengths, weaknesses, and required modifications through thematic analysis. Stakeholders valued SHIFT-SHARE’s visual clarity and comprehensiveness, while identifying changes to make its language more accessible, restructuring risk assessment as a continuous process, adding workforce incentivisation as a core consideration, and reconceptualising the framework’s logic model to represent continuous cycles, terminating loops, and contingency exits. We also propose implementation support tools, including a term of reference document and a workbook of question-based prompts. The SHIFT-SHARE 2.0 addresses stakeholder-identified barriers to TS/S and may help make implementation planning more systematic, responding to the World Health Organization’s calls for TS/S-specific frameworks. Further validation across diverse contexts will be needed to assess its broader utility.

Health systems globally face provider shortages, with deficits of 12.9–18 million expected by 2035 [1-3]. Task shifting and sharing (TS/S), which moves responsibilities from more to less qualified workers, have been proposed as strategies for responding to these challenges [4]. The World Health Organization (WHO) advised nations to develop regulatory and implementation frameworks to scale TS/S for urgent issues [5]. However, despite widespread application [6], practical guidance for its safe, systematic, and sustainable implementation has remained limited. General frameworks like the Practical, Robust Implementation and Sustainability Model (PRISM) [7] and the Consolidated Framework for Implementation Research (CFIR) [8] offer insights, but do not address considerations inherent to TS/S, including competency mapping, risk assessment, and the ethics of redistributing tasks.

To address this gap, we developed the Strategic Healthcare Implementation Framework for Task Shifting, Sharing, and Resource Enhancement (SHIFT-SHARE) [9]. Following scope identification (phase A of framework development) and literature review (phase B), we drew on theories of Lewin [10], Kotter [11], Lean Thinking [12], and Diffusion of Innovation [13], and constructs from frameworks like PRISM [7], CFIR [8], and Calderdale [14] to conceptualise a six-stage cyclical model (phase C) comprising of the following stages:

– environmental scan: assessing needs and readiness;

– priming: task analysis, communication and collaboration;

– risk signal: identifying and mitigating risks;

– capacity building: training and mentorship, support development;

– monitoring and evaluation: quality assurance and outcome measures;

– maintenance and diffusion: sustainability, scalability, and diffusion.

Four principles underpinned all stages: clinical safety, patient-centredness, ethics, and stakeholder feedback [9]. Stakeholder inputs into framework development can make them more relevant and applicable [15]. The CFIR, for example, underwent updates based on user surveys [8] and iterative Delphi exercises with international scholars who helped build consensus on elements of a conceptual framework for TS/S [16]. Recognising that end-users can identify confusing terminology, structural issues, and overlooked considerations, we summarise phases D (stakeholder feedback) and E (framework refinements) [9] in this viewpoint, alongside presenting support tools for our updated framework.

## STAKEHOLDER CONSULTATION IN INDIA

As part of his dissertation, one author (SD) set out to validate and improve SHIFT-SHARE *via* a mixed-methods study in India [17]. The country provided polar contexts (urban to rural) with TS/S use across primary care, mental health, and emergency medicine (EM) and emergency medical services, plus ongoing system-wide reforms incorporating task redistributions [18,19]. This diversity offered an opportunity to surface implementation challenges that are likely relevant across health systems with varying resource levels and regulatory environments. Between November 2023 and September 2024, the researcher (SD) conducted eight focus group discussions (FGDs), gathering feedback on SHIFT-SHARE’s relevance, accessibility, and improvement areas. Participants received a three-page SHIFT-SHARE overview at least one week prior. Each FGD included up to six participants and lasted approximately one hour. Discussions explored readability, relevance to work contexts, enablers and barriers, and perceived merits and concerns. Data was anonymised, albeit with some ethical tensions [20], and was analysed thematically using NVivo, version 15 (Lumivero, Denver, Colorado, USA). Participants included doctors, EM specialists, nurses, paramedics, community health workers, organisational leaders, policy experts, and public health students from six Indian states. Details on methods, participant profiles, and participant quotes are provided separately (Section A of **Online Supplementary Document**).

## FRAMEWORK RECEPTION AND TERMINOLOGY REFINEMENTS

Stakeholders appreciated SHIFT-SHARE’s visual presentation, with its use of colour-coding, numbering, and flow making complex TS/S concepts more understandable. Participants described the framework as ‘very straightforward’ (FGD07-R1: physician, project manager) and ‘conceptually brilliant’ (FGD07-R2: physician, programme manager), highlighting its potential to systematise informal TS/S practices. Accessibility is important, as confusion over terminology can result in inadequate adoption [21] and clear terminology determines correct implementation [22,23]. Stakeholders envisioned SHIFT-SHARE as a ‘tabletop exercise’ (FGD07-R3: physician, management consultant) for teams to discuss intervention design, potentially facilitating TS/S as a human resources strategy, rather than merely a temporary fix [24]. Importantly, stakeholders noted that organisations typically focus on needs assessment, training, and monitoring (stages 01, 04, 05) but often overlook elements in stages 02, 03, and 06, suggesting SHIFT-SHARE may help ensure more comprehensive planning.

However, several terms confused stakeholders. They associated ‘environmental scan’ with ecological, rather than contextual assessment, and instead suggested ‘situational analysis’ as a more intuitive term (FGD02-R1: public health professor; FGD06-R1: research council scientist), particularly given its more common usage in Indian literature [25-29] compared to the North American and Australian contexts where ‘environmental scan’ predominates [30-32]. The distinction between ‘workforce needs’ and ‘workforce capacity’, two distinct human resources planning concepts [33], was perceived as unclear (FGD04-R3: EM department head; FGD07-R1), with ‘workforce requirements’ and ‘workforce capabilities’ preferred. ‘Priming’ proved problematic, with consensus among FGD04 participants associating it with infusion pumps, fruit ripening, or generic planning (FGD04-R1: EM physician, hospital director; FGD04-R3; FGD04-R4: ambulance operations vice-president). ‘Preparation’ was consistently recommended as clearer. Regarding ‘maintenance and diffusion’, there was a shared view among FGD05 participants that ‘diffusion’ was difficult to interpret in a knowledge-sharing context (FGD05-R2 and FGD05-R3: public health students), and the distinction between ‘replicability’ and ‘reproducibility’ was questioned (FGD04-R4) despite alignment with the literature [34-36]. Finally, while the four underpinning considerations were valued, participants felt they got lost in the display and suggested renaming ‘underpinning considerations’ to something more immediately understandable (FGD07-R3; FGD08-R1: public health student). Although these terminology challenges were identified in India, they likely reflect broader difficulties in translating implementation science concepts globally.

## REPOSITIONING RISK ASSESSMENT

Stakeholders within FGD-04 (FGD04-R3; FGD04-R4) argued that conducting needs assessments before identifying major risks could be a wasted effort if rudimentary obstacles emerged late, which is worth considering, as evidence supports that TS/S without early risk assessment compromises patient safety [37]. Participants also emphasised explicitly mentioning risk types beyond clinical safety, including financial risk *via* cost-benefit analysis and worker safety (particularly gender-specific concerns such as restrictions on female staff working night shifts in isolated areas). Legal risks also proved important: FGD06-R1 recounted Kerala state’s experience, where medication prescribing was shifted to public health nurses but later challenged by pharmacy associations and stopped by court order [38,39]. Patients receiving care *via* this TS/S model had to revert to primary health centres due to legal issues, underscoring the relevance of incorporating diverse risk categories early in planning.

## MISSING WORKFORCE INCENTIVISATION

The original SHIFT-SHARE skipped workforce incentivisation considerations, which stakeholders emphasised as critical, suggesting it should extend beyond monetary benefits to include conference attendance, funding for travel, and work breaks (FGD02-R1; FGD07-R2; FGD07-R3). Indeed, extending responsibilities without proportional benefits can negatively influence TS/S attitudes, creating ‘a sense of injustice and resentment’ [40]. Unfair compensation is a documented barrier to TS/S implementation across low- and middle-income countries, and similar concerns have been raised in high-income settings [41,42]. Without appropriate incentives, TS/S risks resistance and unsustainability [35].

## LIMITATIONS OF A CYCLICAL MODEL

Participants challenged SHIFT-SHARE’s singularly cyclical nature. There was agreement within FGD-07 in questioning whether every task shifting necessarily leads to more shifting, given legal liabilities, infrastructural issues, and saturation points (FGD07-R2; FGD07-R3). They suggested a three-option model: continuous cycles, definitive endpoints, or reversals when TS/S proves unsuccessful. The need for an ‘escape route’ was also emphasised (FGD05-R4: nurse, public health student; FGD07-R1), for example, when tasks performed by a new cadre need to be handed back to the delegating cadre or referred to another facility. These suggestions align with theoretical foundations: Nelson’s Clinical Microsystems Theory [43] emphasises that providers must stay connected to referral networks, Contingency Theory [44] argues for tailored approaches to implementation challenges, and Lewin’s Force Field Analysis [10,45] explains that, when constraints counterbalance benefits, continuation may be unfeasible.

## SHIFT-SHARE 2.0 AND SUPPORT TOOLS

**Figure 1:**
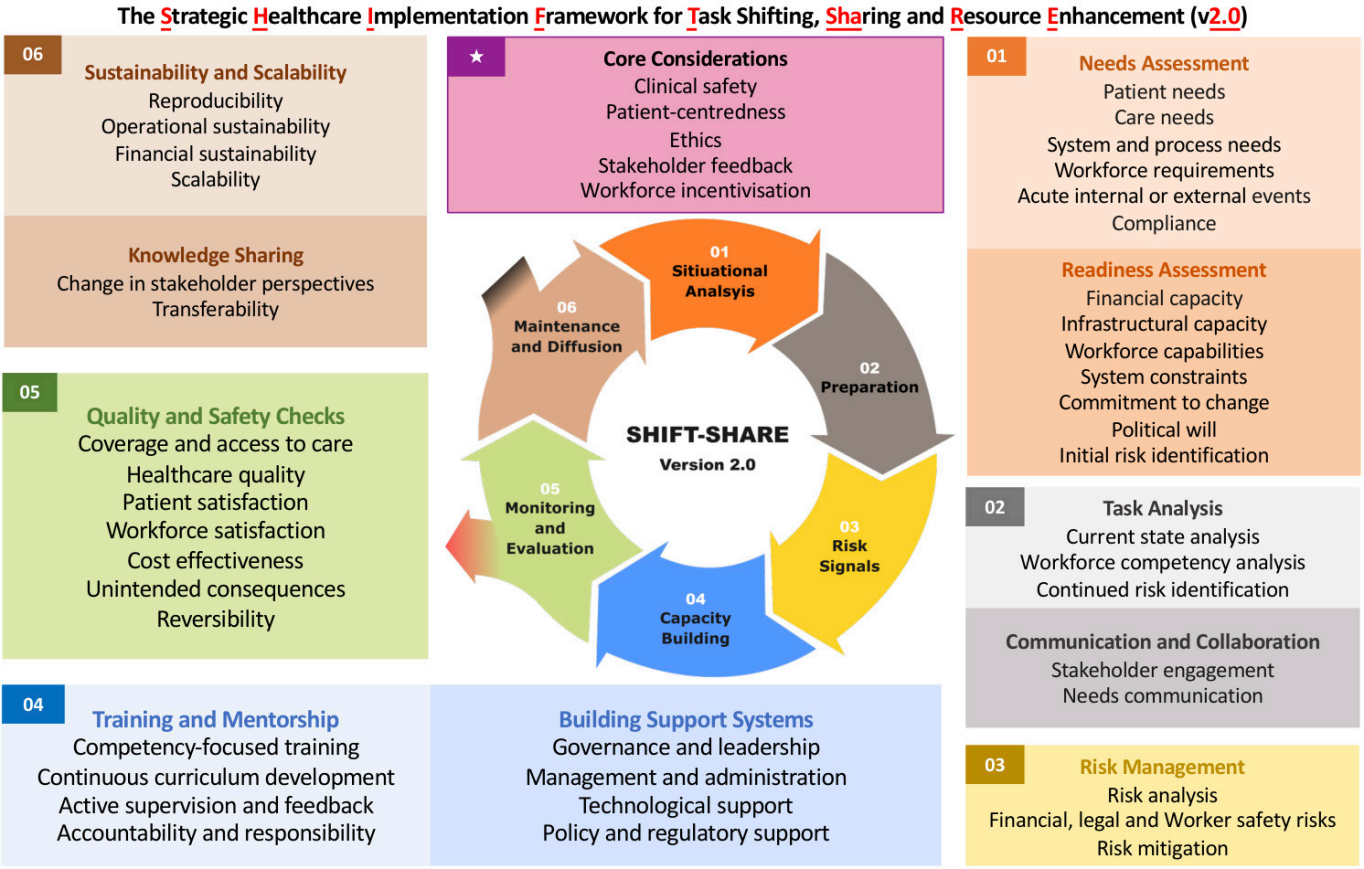
SHIFT-SHARE 2.0 for task shifting and sharing. A six-stage framework guiding the planning and implementation of task shifting and sharing with three pathways. Five core considerations (starred box) underpin all stages. Task shifting or sharing can follow one of three pathways: a) continuous cycle: progression to subsequent TS/S cycles (continuous arrows), b) terminating loop: exit when endpoints are reached (black projection), or c) contingency exit: exit for referrals or reversals (red projection).

Bringing together stakeholder insights, we developed SHIFT-SHARE 2.0 with revised terminology, restructured components and logic models, and new support tools (**Figure 1**). Terminology revisions include: ‘environmental scan’ to ‘situational analysis’; ‘workforce needs’ to ‘workforce requirements’ and ‘workforce capacity’ to ‘workforce capabilities’; ‘priming’ to ‘preparation’; ‘diffusion’ to ‘knowledge sharing’; removing ‘replicability’ while retaining ‘reproducibility’; and ‘underpinning considerations’ to ‘core considerations’ (using distinctly coloured bordered box with star symbol for prominence).

We restructured risk assessment as a three-fold approach: ‘initial risk identification’ during ‘situational analysis’ (identifying ‘deal-breaker’ risks early); ‘continued risk identification’ in ‘task analysis’ stage (recognising certain risks emerge only when examining specific tasks); and ‘risk management’ as a dedicated component following ‘preparation’ (where risks are collated, analysed collaboratively, and mitigated). We now explicitly mention financial, worker safety (including gender-specific aspects), and legal or regulatory risk categories. We added ‘workforce incentivisation’ as a fifth core consideration, joining clinical safety, patient-centredness, ethics, and stakeholder feedback. Workforce incentivisation includes financial incentives, professional development, career advancement, and improved working conditions, with budget implications recommended to be considered from the outset.

The SHIFT-SHARE 2.0 acknowledges multiple pathways, integrating three logic models (**Figure 1**):

− continuous cycles when subsequent TS/S is possible;

− terminating loop when definitive endpoints are reached after one TS/S cycle due to legal constraints or resource limitations (exit from stage 06);

− and contingency exits providing reversal pathways when TS/S becomes ineffective or referrals when care exceeds provider capability (exit from stage 05; after monitoring and evaluation identifies need for exit or referral).

This representation is a more realistic depiction of TS/S implementation realities. To further add utility, we propose two complementary resources. First, a term of reference file (Section B of **Online Supplementary Document**), offering definitions for all framework elements. By including standardised definitions, the terms of reference document can enable or aid proper implementation and knowledge translation [46]. Second, a workbook (Section C of **Online Supplementary Document**), suggesting two to three question-based prompts for each framework element using ‘have you...?’, ‘do you...?’, and ‘can you...?’ formats to prompt reflection throughout the TS/S life cycle. Question-based prompts are widely used to improve communication and practice [47-49], and there is evidence that cues, as such, help stakeholders make better decisions [50].

The SHIFT-SHARE 2.0 represents an evolution from a theoretical model toward a more practical tool, showing the value of stakeholder engagement in framework development. By addressing terminology confusion, repositioning elements, and acknowledging multiple pathways, the updated framework better reflects TS/S’s implementation realities and responds to the WHO’s calls for developing implementation frameworks to expand TS/S. Future work could improve the support tools by creating an interactive digital platform that integrates the terms of reference and prompts, with hyperlinks to complementary open-access tools such as organisational readiness assessment questionnaires [51], RE-AIM templates [52,53], and CFIR codebooks [54]. A one-stop resource like this could host interactive features such as branching decision pathways for risk scenarios or auto-populated checklists linked to each stage, providing direct implementation support to service planners. While our qualitative study identified themes in India, including terminology barriers, risk timing, incentivisation gaps, and pathway limitations, these are challenges in TS/S globally, and further validation studies in other settings are needed to ensure the SHIFT-SHARE’s transferability. The ultimate contribution of the SHIFT-SHARE 2.0 in transforming TS/S from a reactive, poorly documented phenomenon into a strategic approach to improving worker utilisation, health care access, and resilience will depend on implementation fidelity and contextual adaptation.

## Additional material


Online Supplementary Document
