# Supplementary material for: An updated framework for task shifting and sharing: refining the SHIFT-SHARE based on stakeholder feedback from India
Source: J Glob Health. 2026 Mar 20;16:03008. doi: 10.7189/jogh.16.03008 (PMC13002171; doi:10.7189/jogh.16.03008)
Supplement: Online Supplementary Document [file jogh-16-03008-s001.pdf]

Supplement to: Das S, Grant L, Fearon D, Weller D. An updated framework for task shifting and sharing: refining the SHIFT-SHARE based on stakeholder feedback from India. J Glob Health. 2026;16:03008.

SECTION A
Methods and Results from Focus Group Discussions

PART 1: METHODS

This study was conducted as part of author SD's PhD fieldwork in India between November 2023 and September 2024 to validate and refine the SHIFT-SHARE through stakeholder engagement.

Sampling Strategy and Participant Recruitment

Purposive sampling was used, complemented by convenience sampling to recruit participants with relevant TS/S knowledge and experience. SD was based at Lifeline Foundation, an Indian non-governmental organisation (NGO) in Vadodara, Gujarat*, which facilitated connections with organisations implementing TS/S-based programmes. Snowball sampling expanded the sample by asking enrolled participants for referrals to others engaged in TS/S work. The following criteria were used for recruitment:

- **Inclusion criteria**: Healthcare providers, managers, trainers, academics, policymakers, researchers, funders, students, or beneficiaries involved in or affected by TS/S; aged ≥18 years; capable of providing informed consent; from any gender, income level, or regional background; from private or public sectors.
- **Exclusion criteria**: Those involved in task shifting/sharing between human resources for health at the same professional levels; unwilling or unable to provide written informed consent.

Informed Consent Process

Potential participants received three documents via email one week before scheduled discussions: (1) Participant Information Sheet explaining study background, purpose, participation requirements, and data handling; (2) Consent Form recording agreement to enrol; (3) Participant Data Form collecting work details to assess eligibility. All documents were available in English and Hindi. Participants could complete forms electronically or in person before data collection. After signing, each received a copy of the signed consent form.

---

* Lifeline Foundation. 2025 Available: https://www.emsindia.in

FGD Design

- **Rationale and pre-reading materials:** Focus group discussions (FGDs) were selected to assess how organisations align with SHIFT-SHARE's philosophy and gather expert feedback. One week before scheduled FGDs, participants received a three-page overview of SHIFT-SHARE, including its illustration and description, allowing familiarisation with the framework's structure, terminology, and intended applications.
- **Topic guide:** A topic guide was developed, structured around: readability and clarity; relevance and applicability; underpinning features; anticipated enablers and barriers; and perceived merits and concerns. Questions included: "How would you rate the overall readability of the model?", "Do you think the language or terminology used is clear?", "Which stages would be most critical to effective TS/S?", "Is there any other stage or process the model has missed out?", and "What are your thoughts on the model being represented as a cycle?"
- **FGD conduct:** Eight FGDs were conducted between November 2023 and September 2024. Seven were in-person at participant offices; one was virtual via Microsoft Teams. FGDs included between 3–6 participants: one had six participants, four had five participants, two had four participants, and two had three participants (mini-Focus Groups when larger groups could not be assembled). FGDs lasted an hour on average (range: 60-76 minutes). Open-ended prompts were used to encourage sharing, discussions were moderated to maintain focus, and non-directive language was employed. All discussions were audio-recorded with consent.

Data Processing and Analysis

- **Transcription and translation:** English recordings were transcribed word-for-word. For Hindi or Gujarati recordings, SD listened and directly translated whilst typing into English, checking draft transcripts against field notes. Personal identifiers were removed, and quotes were vetted to maintain confidentiality.
- **Member checking:** Complete transcripts were shared with participants who requested review. After visits, the researcher sat with participants to go through notes and reflect on observations, allowing them to correct details or reword statements.
- **Analytical approach:** Thematic analysis was conducted on FGD transcripts focused on direct feedback on the framework, examining visual presentation and comprehensiveness; terminology clarity; suggested modifications; and practical application considerations. Analysis followed Braun and Clarke's approach: familiarising with data, conducting initial coding, searching for themes, reviewing them, defining them, and reporting findings. Field notes and summary notes were maintained to capture context and identify key themes.

PART 2: RESULTS

Participant overview: Of 79 total participants recruited across all methods (semi-structured interviews and participant observations), 35 were recruited in eight FGDs designed to gather feedback on SHIFT-SHARE. These were from six Indian states, from across urban, semi-urban, and rural contexts, working in emergency medical services, primary care, mental health, telemedicine, and tertiary care. The excerpts represent 23 participants, whose comments have been included in the paper, addressing terminology, structure, accessibility, and applications. Other participants contributed confirmatory comments or discussed other aspects not directly relevant to framework refinement. Participants are coded as FGD[number]-R[number], where the first number indicates which FGD they were in, and the second number indicates their respondent number within that FGD.

Participant Code	Profile	Organisation/Affiliation
FGD01-R1	Deputy chief executive officer (CEO)	A Gujarat-based NGO training AYUSH doctors in pre-hospital emergency response across multiple states
FGD02-R1	Assistant Professor, Public Health	A private University in Gujarat
FGD02-R2	Assistant Professor, Health Systems and Policy	A private University in Gujarat
FGD03-R1	Coordinator	An NGO implementing community worker-led screening and health education in rural Gujarat
FGD03-R2	Managing Trustee	An NGO implementing community worker-led screening and health education in rural Gujarat
FGD04-R1	Emergency Medicine Physician, Director	A private hospital in Kerala
FGD04-R2	Emergency Medicine Physician, Director	A private hospital in Delhi
FGD04-R3	Physician, Head of Emergency Medicine Department	A teaching university hospital in Tamil Nadu
FGD04-R4	Emergency Medicine Physician, Vice President (Operations)	A Telangana-based private ambulance service provider using remotely guided paramedics across multiple states
FGD05-R1	Public health student	A private University in Gujarat
FGD05-R2	Public health student	A private University in Gujarat
FGD05-R3	Public health student	A private University in Gujarat

FGD05-R4	Nurse and public health student	A paramedical teaching institute in Gujarat training doctors for rural emergency care
FGD06-R1	Scientist	A national-level research council based in Delhi
FGD06-R2	Scientist	A national-level research council based in Delhi
FGD06-R3	Project Scientist	A national-level research council based in Delhi
FGD07-R1	Physician, Project Manager	A Delhi-based NGO working towards improving health access across different states
FGD07-R2	Physician, Programme Manager	A Delhi-based non-profit research institute with a focus on digital health and capacity building across India
FGD07-R3	Private Physician, Management Consultant	Independent consultant based in Delhi
FGD08-R1	Public health student	A private University in Maharashtra
FGD08-R2	Public health student	A private University in Maharashtra
FGD08-R3	Public health student	A private University in Maharashtra
FGD08-R4	Physiotherapist and public health student	A private University in Maharashtra

Participant Quotes

1. FRAMEWORK ACCESSIBILITY AND POTENTIAL

VISUAL PRESENTATION AND READABILITY: Participants consistently appreciated the framework's visual presentation, noting that its design features enhanced accessibility and comprehension.

Quote 01 (FGD08-R1):

> *"The readability is easy because it is colour coded, it has numbers, so, yeah, it's easy to go through... We don't have to find where does it start, where does it end. It's quite clear... how the flow goes."*

Quote 02 (FGD01-R1):

> *"I could understand what you meant... When I look at the circle, circular structure, and compare it with different titles [in the boxes], considering colours are same, it's good."*

Quote 03 (FGD07-R1):

> *"I think the model...makes sense. Yeah, it's talking about how do you do TS/S, and it's walking you through different stages of processes. It's very straightforward. I like the representation also."*

Quote 04 (FGD08-R2):

> *"Very clear as language used is quite understandable. I will rate 10 out of 10."*

Key Finding: Colour-coding, numbering, and circular layout improved accessibility. The visual flow helped frontline HRH, managers, and community stakeholders grasp TS/S concepts through shared understanding.

COMPREHENSIVENESS AND DEPTH: Participants appreciated that SHIFT-SHARE maintained depth whilst avoiding unnecessary complexity.

Quote 05 (FGD05-R1):

> *"It's not very complex. It details the things required for going ahead with [TS/S]. All aspects, say number four, training and mentorship, list each small detail... competency, risk assessment... It's really good."*

Quote 06 (FGD07-R2):

> *"Overall, it's a very good model. I really appreciate that you've developed this...it's a very good way of getting task shifting done. Conceptually, it's brilliant. It's just about piloting it and figuring out what are key important points are, and what are not."*

Quote 07 (FGD03-R2):

> *"The model which you had made is going particularly very nice...divided and all, which is really required for all the NGOs to work on [TS/S]. So, this is a really nice model, and we don't have any questions regarding the same."*

Quote 08 (FGD04-R1):

> *"Looking at it, it's a very good model. All this kind of exercise is happening [in reality], but we are not naming or picturing (it) this way."*

Key Finding: The framework captures necessary aspects for TS/S implementation, with each stage breaking down components into actionable considerations. Experienced practitioners recognised that whilst TS/S already occurs in practice, SHIFT-SHARE provides structured qualification of these informal practices.

POTENTIAL APPLICATIONS**:** Stakeholders identified multiple direct uses for SHIFT-SHARE, especially its potential to plan, document, and standardise mechanisms in TS/S that are currently informal.

Quote 09 (FGD07-R3):

> *"I think this framework, this thought model...will be a good thought exercise, or a tabletop exercise for people to actually sit literally on a table and discuss what can be done, what cannot... that's something I feel is of good value."*

Quote 10 (FGD04-R2):

> *"This could be a reference for others...It also brings consistency if you're doing shifting as per one model instead of doing (it) as per different models, that's important."*

Quote 11 (FGD07-R2):

> *"When we develop concept notes...proposal letters or...reports, we don't have a standardisation. I ask one team member to do it, they'll do it. I'll ask other...they'll do it in their preference.... Your framework (has a) lot of utility in building structured methodical proposals and reports."*

CRITICAL OBSERVATION - GAPS IN CURRENT PRACTICE

Quote 12 (FGD07-R2):

> *"Most would focus on need assessment and...training...monitoring evaluation...number one, number four and number five (stages of SHIFT-SHARE), but number two, three, six, most miss out on these aspects."*

Quote 13 (FGD02-R2):

> *"In operations management of (a) hospital or research itself, I think considering all these tasks...that they have to do some sort of need assessment, and identify the gap, present the report, and then the hospital is ready to (do TS/S), same goes for the programmes also."*

Quote 14 (FGD07-R2):

> *"It will help us improve ourselves...gives you that structure to...put things in a certain logical order... so that they will be understood properly. I think that gives it that structure that is missing."*

Key Finding: SHIFT-SHARE could reshape TS/S from poorly documented practices to a systematic approach. Stakeholders identified use as a tabletop exercise tool, documentation template, and standardisation reference. Participants noted organisations typically focus on needs assessment, training, and monitoring (Stages 1, 4, 5) whilst overlooking Stages 2, 3, 6.

2. STAKEHOLDER FEEDBACK ON FRAMEWORK TERMINOLOGY

CONFUSION AROUND 'ENVIRONMENTAL SCAN': The term 'Environmental Scan' caused consistent confusion amongst participants. Multiple stakeholders stated that 'environmental' in public health contexts typically refers to the ecological or physical environment, not the organisational assessment as intended.

Quote 15 (FGD02-R1):

> *"...in (the) first stage, it's a kind of situation analysis that you are doing, right? I don't perceive this... particular word (environmental). So, instead, just scan, maybe? If not scan, maybe situation analysis kind of simple word, which is easily understandable."*

Quote 16 (FGD06-R1):

> *"We can add situational analysis here instead of environmental scan. It's not related to the health...in public health, environment (can) mean something else."*

Quote 17 (FGD07-R1):

> *"... Environmental scan. Is it to do with (the) environment?"*

Key Finding: Participants suggested 'Situational Analysis' as a more intuitive alternative to convey the stage's intent.

CONFUSION REGARDING 'WORKFORCE NEEDS' AND 'WORKFORCE CAPACITY': Although 'Workforce needs' and 'Workforce capacity' were included under different assessment categories (needs versus readiness), participants found the distinction unclear.

Quote 18 (FGD04-R3):

> *"What is the difference between workforce needs and workforce capacity. I thought that (was) confusing. I thought both mean the same. There's a lot of overlap between (both)."*

Quote 19 (FGD07-R1):

> *"Some confusion, I think, between some terms in readiness assessment. I'm not sure what (the) difference is between workforce needs and workforce capacity?... I don't know whether you mean the same thing here. I would like more clarity on (them)."*

Key Finding: Whilst intended to distinguish between gaps/requirements (what skills are missing, what support is needed) and current capabilities (existing competencies, staff availability, workload capacity), users saw overlap between these terminologies.

***CONFUSION AROUND THE TERM 'PRIMING'*:** The word 'Priming' was identified as problematic across multiple FGDs. Participants were unsure about its meaning and associated it with different things.

Quote 20 (FGD04-R4):

> *"No idea what that means. My idea is that we prime the infusion pump."*

Quote 21 (FGD04-R1):

> *"Nowadays, it's widely used in terms of planning meetings. Or just like mango priming from yellow to red (laughs)."*

Quote 22 (FGD04-R3):

> *"... these guys are all into businesses...they have different ideas. My idea of priming, priming of a dream? You should find some other term."*

Quote 23 (FGD07-R2):

> *"Why don't we just call it a preparation or something? Not sure whether medical folks will really understand the term priming in this context... Something to do with preparation would be more suitable, that's just my opinion."*

Quote 24 (FGD07-R1):

> *"(Laughs) Reminded of prime numbers from maths, and I don't suppose this has to do anything to do with mathematics. I agree... we could have something to do with preparation."*

Key Finding: The term did not convey the preparatory stage as intended (analysing current tasks, mapping competencies, engaging stakeholders before actual TS/S). Many recommended replacing 'Priming' with 'Preparation'.

CLARITY ON TERMS IN 'MAINTENANCE AND DIFFUSION' STAGE: Stakeholders reported confusion about certain terms in the sixth stage.

Quote 25 (FGD05-R2):

> *"(The) point that is sustainability, scalability, and then is diffusion. For some people, it will be difficult to understand what actually diffusion is like... (it) will take more time to understand that particular component."*

Quote 26 (FGD05-R3):

> *"The word diffusion must be modified into something that will match properly, and it will emphasise clearly...the focusing on the sharing (of) knowledge, transferring the information. Rest points are clearly understandable."*

Quote 27 (FGD04-R4):

> *"So, what is this? Replicability and reproducibility, what's different?"*

Quote 28 (FGD07-R1):

> *"What's the difference between replicability and transferability? I felt similarly about what you mean by sustainability, scalability... These words we do come across in... environmental and SDG goals about sustainability and all of that. I don't know whether you mean the same thing here. I think that's something I would like more clarity on."*

Key Finding: ‘Diffusion’ did not clearly communicate knowledge sharing and spreading. ‘Sustainability’ and ‘Scalability’ were associated with environmental sustainability and Sustainable Development Goals contexts. Distinctions between ‘Replicability’ and ‘Reproducibility’ were unclear.

IMPORTANT CONTEXT

Quote 29 (FGD07-R2):

> *“Diffusion... very important aspect, and we don’t focus on that. We do a lot of work that is ...done only once, and then, end of funding, or interest usually tapers down, so you don't sustain it. So, focusing on sustainability and scaling, and diffusion is a very important phase of task shifting, which most organisations don’t do. With the current political climate, there’s a drastic drop in funding availability. When that happens, automatically, you don't think about diffusing, don’t think about scaling at all, because you just want to finish your project, write a report, and get done with it. Because you know you won’t, or you might not get a grant again. So, I think it’s two-way. If there’s no funding, there’ll be no interest in scalability or diffusion. But if there’s also no planning of scalability and diffusing, how do you go back to a funder and ask for more funding?”*

Key Finding: Despite terminology issues, participants recognised how often projects are initiated but not sustained or scaled due to budget limitations or loss of interest, suggesting that whilst the terms need refinement, the underlying concepts are key to TS/S.

MIXED UNDERSTANDING OF ‘UNDERPINNING’ CONSIDERATIONS: There was a small but key feedback on representing the 'Underpinning considerations' in the framework diagram.

Quote 30 (FGD07-R3):

> *“Clinical safety is a very important point of consideration in all shifting, sharing. If it isn’t clinically safe, you should not engage. Similarly, patient-centeredness... It should be ethical, and stakeholder feedback... Can you highlight it in a better way? I would normally start from here (pointing to Stage 1: Environmental Scan) and then go here (pointing to Stage 2: Priming). I think (underpinning considerations) gets lost out (in the display).”*

Quote 31 (FGD08-R1):

> *“There’s one term called Underpinning Considerations. I feel that not many of us can understand it in the first go. It could also be simplified. The points (in it) are understandable, but like what does the title (say)?”*

Key Finding: Participants appreciated the inclusion of underlying central considerations (clinical safety, patient-centredness, ethics, stakeholder feedback) but noted the block gets lost

in visualisation and the title could be simplified. The word ‘Underpinning’ was unclear, though its elements were understandable.

3. SUGGESTED STRUCTURAL CHANGES TO SHIFT-SHARE

POSITION OF RISK ASSESSMENT: In original SHIFT-SHARE, ‘Priming’ (task analysis, communication and collaboration) comes before ‘Risk Signal’ (risk identification and mitigation). However, participants across FGDs stated that conducting risk assessments after needs and readiness assessments, task analysis, and stakeholder engagement may result in futile effort if major risks are identified late in the planning process.

Quote 32 (FGD04-R3):

> *“You’re doing a needs assessment first and then only looking at risk. Now, if I feel something is very risky, why would I actually embark on a needs assessment? Suppose you say that I want all nurses to learn this skill. You have done the hard work...and finally somebody comes and tells you it’s a very risky proposition. All your hard work gets wasted. You should...look at risks of what you are embarking upon when you actually start.”*

Quote 33 (FGD04-R4):

> *“FGD04-R3 is right. I think the risk assessment should come first. Before you take up the whole process.”*

Quote 34 (FGD06-R1):

> *“I think the risk signal stage should come before priming. You could combine task analysis and risk assessment into one stage and have something like communication and collaboration as a different stage after it.”*

Quote 35 (FGD06-R2):

> *“Yes, also agree. I think it makes sense to actually have task analysis and risk assessment together.”*

Quote 36 (FGD07-R2):

> *“Readiness assessment and communication and collaboration happen together, is what I’d say. Risk assessment is also something that might happen at readiness assessment stage...not sure why it’s after. Maybe do risk signals before communicating and collaborating? By the time you already started something, and (if) you haven’t evaluated risks, and then, after that, you realise there are risks, essentially, you are just wasting a lot of time, money, and effort. I think the risk signal should come before priming.”*

Quote 37 (FGD08-R3):

> *“... Risk assessment should be before the task analysis, I think. First, you identify the risk, then you can think of delegating the task.”*

Quote 38 (FGD03-R1):

> *"I think first, environmental scan, then after we have to go for risk signals, then after priming or capacity building. So, after risk we are ready to do whatever...should be done. Risk signal should be on second position."*

Key Finding: Participants saw logic in blending risk assessment with previous stages ('Readiness assessment' or 'Task Analysis'), as one cannot assess risks without understanding whether providers have the training and capacity to do tasks or without examining specific tasks being shifted or shared. They advocated mapping risks before delegating tasks or communicating with stakeholders about TS/S.

EXPLICIT MENTION OF RISK TYPES: Participants also suggested mentioning types of risks that service planners might want to consider.

FINANCIAL RISKS (Conversation between FGD04-R4 and FGD04-R2):

Quote 39 (FGD04-R4):

> *"In your risk assessment, your cost-benefit ratio should come because it's a financial risk to the institution that's doing (TS/S)."*

Quote 40 (FGD04-R2):

> *"Yes, I think you can add there. That's a good idea."*

WORKER SAFETY CONSIDERATIONS (Conversation among FGD04-R4, FGD04-R3, FGD07-R2, and FGD07-R3):

Quote 41 (FGD04-R4):

> *"One thing I feel is not covered is safety. You (have) clinical safety, but worker safety?"*

Quote 42 (FGD04-R):

> *"Like, do not send the lady nurses or the lady paramedics in the ambulance after 8 o'clock for patient transfers."*

Quote 43 (FGD04-R4):

> *"I couldn't post the lady EMT at the hospital, just because, at night shifts, it becomes really dark, deserted. There's an industrial area, a lot of unsafe areas."*

Quote 44 (FGD07-R2):

> *"Workforce safety is a big consideration, especially in rural areas. If you're working with CHWs and they're in rural areas...going from house to house, meeting family to family, they're travelling in remote locations. Is it safe for them to actually do that? If it*

> *is not, how do you build safety? And how do you prepare them in case of emergency situations? Those considerations are important."*

Quote 45 (FGD07-R3):

> *"I agree."*

LEGAL RISKS

Quote 46 (FGD06-R1):

> *"But legal risks, the term legal, is very important. Reason I'm saying is in Kerala...they started (TS/S) long before... (junior public health nurses) giving medicines for NCDs, right? Diabetes and hypertension they managed at sub-centre level. The pharmacology association, pharmacists, went to High Court of Kerala, got a stay on that. They said drugs can only be provided by pharmacists, not by a nurse. So, now, because pharmacists are not available at sub-centre level, it all shifted back to the PHC. All patients who were actually getting their treatment...which was task shifted there, had to (reverse) back because of legal issue."*

Key Finding: Participants suggested mentioning financial risks, worker safety considerations (including gender-specific aspects), and legal risks. Particular emphasis on 'Legal risks' based on Kerala policy experience, where TS/S of medicine prescribing to public health nurses was legally challenged and stopped by pharmacy associations.

WORKFORCE INCENTIVISATION AS A MISSING CONSIDERATION**:** Stakeholders pointed out that incentivisation, a key determinant of provider motivation and performance, is missing in SHIFT-SHARE.

Quote 47 (FGD02-R1):

> *"What monetary or non-monetary benefits workforce will be getting? You can incorporate (incentivisation) in whichever stage...along with mentorship, we can have that component of advantages."*

Quote 48 (FGD06-R3):

> *"Add incentives also somewhere...your framework has potential to give it order, some direction and structure, which is great."*

CONVERSATION (FGD07-R3 and FGD07-R2):

Quote 49 (FGD07-R3):

> *"Money is also important...if (people) are required to do more tasks than they were initially supposed to do, are you giving money to do it properly...(that) should be in your*

> *underpinning consideration, incentivisation...if you don't have incentivisation planning, it's not going to work out."*

Quote 50 (FGD07-R2):

> *"Yes, yes, I absolutely agree. Incentivisation has to be given a thorough thought if you're planning to do that shifting sharing...can't do it without proper incentivisation."*

Quote 51 (FGD07-R3):

> *"And incentivisation doesn't necessarily always mean money, let me clarify. It could be other ways...we used to do it in a hospital, so junior residents, we weren't able to give more money...but gave privileges. They go to more conferences, events. We form funding opportunities for their travel...they get break from work. What opportunities you're actually giving...is extremely important."*

Quote 52 (FGD08-R4):

> *"I think incentive methods...that (if) they are getting involved, they should also benefit from it. When they are satisfied, they will provide the right care, and they will make sure that they have the right skillset. So, making sure that they are satisfied and taking their needs would be...helpful."*

Key Finding: Participants emphasised that incentivisation should not be optional. It extends beyond monetary compensation (salary increases, allowances) to professional development opportunities (conferences, events), career advancement, breaks, and other advantages that lead to satisfaction, motivating people to undertake skill-building and provide better care.

CRITIQUE OF SHIFT-SHARE'S CYCLICAL NATURE

Participants questioned whether all TS/S-based interventions necessarily follow a cycle, suggesting that SHIFT-SHARE could benefit from considering alternative logic models.

Quote 53 (FGD07-R3):

> *"Why (do) you have a cycle? Not necessary that every task shifting will result in more shifting, right? So, I don't know whether you want to show a cycle or whether you want to...branch it out. One route could be a cyclic route, and one could just end...because a lot of shifting can actually just end after it's done, there's only so much scope. Then there's legal liability, infrastructural issues, there's a limit, right? There's a saturation point at which you cannot do more task shifting."*

Quote 54 (FGD07-R2):

> *"It might end...might also go back. I think bifid (structure) is a good option. I also think whether you can show a model where you have three options. One option is, of course, it's starting and ending. So, that is the whole task shifting, sharing, process. Other option is that you go back to main starting point...the cycle that you have. And third option,*

> *when it's interrupted or being reversed, because that's also a practical reality, not all task shifting actually happens successfully."*

REFERRAL PATHWAYS AND CONTINGENCY PLANNING

Quote 55 (FGD05-R4):

> *"The ethical part is the referral pathway. So, example, it depends on individuals, how well they can contain ethics, like patients may see or know that particular health worker is not suitable for them, or (health worker) may not be good to attend to them as such. So, I see that referral pathway means if I'm seeing a patient and the patient is beyond my capability, then I have to do a referral, rather than trying to make attempt to you know (do) something that I don't know."*

Quote 56 (FGD07-R1):

> *"Yeah, I still think what it should include is the loop of actually reversing it or what happens if something goes wrong, you know, an escape route planned? I think you should have talks about (being) reversible. What if it doesn't work, what is the escape plan?"*

Key Finding: Participants noted TS/S could naturally end due to legal, logistical, or infrastructural limitations, and suggested building multiple pathways to represent different outcomes. They raised considerations about referral pathways and contingency planning, emphasising defined patient pathways and "rollback" options when TS/S becomes unworkable, suggesting the need for acknowledging both cyclical continuation and definitive endpoints in SHIFT-SHARE.

4. SUGGESTIONS FOR ENHANCEMENT TOOLS AND RESOURCES

Stakeholders suggested that SHIFT-SHARE's practical utility could be increased through the development of supporting resources and integration with existing tools.

Quote 57 (FGD04-R1):

> *"I think you can have some hyperlinks around this... If you click...some explanation regarding what it discusses should be hyperlinked...explanation will give more clarity."*

Quote 58 (FGD07-R3):

> *"So, maybe if tools are available...perhaps hyperlink? If I click on risk assessment, it takes me to a risk assessment tool that already exists. So, when I'm doing a tabletop exercise and I'm following your model and when I'm at (the) risk assessment stage...brilliant, how do I do it? You click and you go to this readily available tool, which will help you do the risk assessment."*

QUESTION-BASED PROMPTS

Quote 59 (FGD07-R3):

> *"Have those templates that you could use to talk to each other and brainstorm, those (are) very useful. So, if a person who's new to TS/S, if he is planning a service...if you're doing readiness assessment and you're interested in understand infrastructural capacity...have a question that asks what sort of infrastructure exists...what additional infrastructure would be required to do TS/S ...Just make in the form of a question or a brief statement for people to use it more practically."*

Key Finding: Participants envisioned an interactive version with detailed explanations, practical tools (such as risk assessment matrices), and question-based prompts to guide users through each element, enhancing SHIFT-SHARE's practical utility.

SECTION B

SHIFT-SHARE 2.0 Terms of Reference Table

The following table explains elements of SHIFT-SHARE 2.0, the updated framework for TS/S. Rows are colour-coded to match the corresponding illustrations in the framework diagram.

Name	Explanation
**Core Considerations**	
Clinical safety	TS/S must be done only if it is clinically safe to do so. All clinical standards must be maintained in the process. Providers with fewer qualifications or training must be able to perform tasks safely. Patient harm should be prevented.
Patient-centredness	Keep patients' needs at the centre of all TS/S. Care must respond to patient preferences as much as possible.
Ethics	Keep appropriate ethical boundaries in mind when employing TS/S. Respect patient autonomy and fairness.
Stakeholder feedback	Take input from all relevant stakeholders regularly, such as patients, providers, administrators, and policymakers. Use this feedback to guide improvements.
Workforce incentivisation	Think of rewards to motivate providers taking on new roles. Use both financial incentives (salary, allowances) and non-financial rewards (professional development, career pathways). Recognise people who are involved in TS/S.
**Stage 01: Situational Analysis**	
Needs Assessment	
Patient needs	Evaluate what patients need. Identify service gaps, such as to do with access, that TS/S could address. Focus on patients' perspectives.
Care needs	Analyse which treatments and support services are needed. Determine which aspects of care could be redistributed among different providers.
System and process needs	Examine workflow and operational requirements that may need changes.
Workforce requirements	Identify what skills staff need. Determine how many personnel are required. Define capabilities needed for effective care.
Acute internal or external events	Consider sudden internal challenges like funding shortages or staff strike, or external crises such as pandemics that may necessitate TS/S.
Compliance	Ensure whether planned TS/S meet local requirements or institutional regulations and policies.

**Readiness Assessment**	
Financial capacity	See whether there is enough funding for implementation. Consider costs for training, supervision, and sustainability.
Infrastructural capacity	Check whether the physical resources and equipment needed to support TS/S are available.
Workforce capabilities	Evaluate skills, knowledge, and aptitudes of staff who will take on new responsibilities under TS/S.
System constraints	Identify limitations, including institutional and regulatory barriers, that may affect TS/S.
Commitment to change	Check staff willingness to adopt to change. Understand whether there is leadership support for TS/S.
Political will	Gauge support from decision-makers and stakeholders with direct influence. Identify who might support or oppose.
Initial risk identification	Do preliminary risk assessment before detailed planning begins.
**Stage 02: Preparation**	
**Task Analysis**	
Current state analysis	Examine the specific activities to be shifted or shared to see whether TS/S is appropriate. See how these tasks are currently done. Document existing workflows, responsibilities, and outcomes.
Workforce competency analysis	Map staff skills and knowledge. Note training gaps. Determine what additional skills are needed.
Continued risk identification	Keep identifying potential risks as planning progresses. Focus on specific tasks to be shifted or shared.
**Communication and Collaboration**	
Stakeholder engagement	Engage with all stakeholders to build understanding and support. Ensure representation from different groups, including practitioners, administrators, and patient representatives.
Needs communication	Explain why TS/S is needed. Set expectations. Convey benefits to build support.
**Stage 03: Risk Signals**	
**Risk Management**	
Risk analysis	Identify and examine potential risks thoroughly. Consider clinical, financial, workforce safety, and legal risks.
Risk mitigation	Come up with strategies to minimise risks. Establish protocols early.
**Stage 04: Capacity Building**	
**Training and Mentorship**	
Competency-focused training	Through training for specific skills, develop confidence of staff who will take on new roles. Focus on practical knowledge.
Continuous curriculum development	Update content regularly. Adapt based on feedback.

Active supervision and feedback	Give regular oversight to maintain quality and safety during TS/S. Give constructive feedback to healthcare providers.
Accountability and responsibility	Define roles and decision-making authority clearly. Establish who is responsible for outcomes.
**Building Support Systems**	
Governance and leadership	Set up regulatory oversight mechanisms. Identify leaders for implementing and sustaining TS/S. Set up committee for problem-solving.
Management and administration	Develop systems and workflows for reporting and managing people involved in TS/S.
Technological support	Provide healthcare providers with appropriate technologies.
Policy and regulatory support	Align organisational policies with TS/S. Protect staff with additional roles.
**Stage 05: Monitoring and Evaluation**	
**Quality and Safety Checks**	
Coverage and access to care	Build mechanisms to measure outcomes and impacts of TS/S. Measure service availability. Track service utilisation rates.
Healthcare quality	Evaluate clinical outcomes. Check adherence to standards.
Patient satisfaction	Study experiences of patient. Take feedback on their preferences and their perceptions of care from different providers.
Workforce satisfaction	Monitor staff experiences and workload levels. Keep a track of how fulfilled and rewarded staff feels in new roles.
Cost effectiveness	Compare resource use against outcomes of TS/S, for example, by calculating cost-benefit ratios.
Unintended consequences	Track unexpected effects and outcomes, both positive and negative.
Reversibility	See if there are instances of TS/S reverting to care as usual. Plan how to return tasks to original providers if TS/S is deemed appropriate.
**Stage 06: Maintenance and Diffusion**	
**Sustainability and Scalability**	
Reproducibility	Test if successful TS/S can be duplicated in the same context or environment.
Operational sustainability	Make sure TS/S-based systems continue to function well. Allocate resources for long-term operation.
Financial sustainability	Look for consistent funding sources. Plan beyond initial implementation or pilots of TS/S.
Scalability	Assess if TS/S-based service can expand to larger populations.
**Knowledge Sharing**	
Change in stakeholder perspectives	Document how attitudes evolve over time. Track understanding among staff and patients about TS/S.

Transferability	See how lessons can be adapted to similar contexts yield comparable results. Consider how approaches can work in different settings or clinical contexts.

References used to define these terms

Cavanagh, S, and K Chadwick. 2005. "Health Needs Assessment: A Practical Guide." National Institute For Clinical Excellence. 2005.

Damschroder, Laura J., Caitlin M. Reardon, Marilla A. Opra Widerquist, and Julie Lowery. 2022. "The Updated Consolidated Framework for Implementation Research Based on User Feedback." Implementation Science 17 (1): 75. https://doi.org/10.1186/s13012-022-01245-0.

Das, Shukanto, Liz Grant, and Genevie Fernandes. 2023. "Task Shifting Healthcare Services in the Post-COVID World: A Scoping Review." PLOS Global Public Health 3 (12): e0001712. https://doi.org/10.1371/journal.pgph.0001712.

Das, Shukanto, Liz Grant, and David Weller. 2024. "Conceptualisation of the SHIFT–SHARE: A New Strategic Healthcare Implementation Framework for Task Shifting, Sharing and Resource Enhancement." Global Implementation Research and Applications, July. https://doi.org/10.1007/s43477-024-00132-8.

Expert Panel on effective ways of investing in Health (EXPH). 2019. "Task Shifting and Health System Design." https://health.ec.europa.eu/system/files/2019-11/023_taskshifting_en_0.pdf.

Feldstein, Adrianne C., and Russell E. Glasgow. 2008. "A Practical, Robust Implementation and Sustainability Model (PRISM) for Integrating Research Findings into Practice." Joint Commission Journal on Quality and Patient Safety 34 (4). https://doi.org/10.1016/S1553-7250(08)34030-6.

Galli, Brian Joseph. 2018. "Change Management Models: A Comparative Analysis and Concerns." IEEE Engineering Management Review 46 (3). https://doi.org/10.1109/EMR.2018.2866860.

Glasgow, Russell E., Samantha M. Harden, Bridget Gaglio, Borsika Rabin, Matthew Lee Smith, Gwenndolyn C. Porter, Marcia G. Ory, and Paul A. Estabrooks. 2019. "RE-AIM Planning and Evaluation Framework: Adapting to New Science and Practice with a 20-Year Review." Frontiers in Public Health. https://doi.org/10.3389/fpubh.2019.00064.

Harvard Business School Online. 2019. "How to Do a Cost-Benefit Analysis & Why It's Important." Business Insights. September 5, 2019. https://online.hbs.edu/blog/post/cost-benefit-analysis.

Hooper, Judith, and Phil Longworth. 2002. "Health Needs Assessment Workbook." London. http://healthimpactassessment.pbworks.com/f/Health+needs+assessment+workbook+-+HDA+England+-+2002.pdf.

Kotter, John P. 2007. "Leading Change: Why Transformation Efforts Fail." Harvard Business Review. https://doi.org/10.1007/978-1-137-16511-4_7.

Langley, GL, KM Nolan, TW Nolan, CL Norman, and LP Provost. 2009. The Improvement Guide: A Practical Approach to Enhancing Organizational Performance. 2nd ed. San Francisco: Jossey-Bass Publishers.

Monden, Yasuhiro. 1983. Toyota Production System : Practical Approach to Production Management.

Okoroafor, Sunny C., and Christmal Dela Christmals. 2023. "Task Shifting and Task Sharing Implementation in Africa: A Scoping Review on Rationale and Scope." Healthcare (Switzerland). https://doi.org/10.3390/healthcare11081200.

Orkin, Aaron M., Sampreeth Rao, Jeyasakthi Venugopal, Natasha Kithulegoda, Pete Wegier, Stephen D. Ritchie, David VanderBurgh, Alexandra Martiniuk, Fabio Salamanca-Buentello, and Ross Upshur. 2021. "Conceptual Framework for Task Shifting and Task Sharing: An International Delphi Study." Human Resources for Health 19(1):61. https://doi.org/10.1186/s12960-021-00605-z.

Rausand, Marvin, and Stein Haugen. 2020. Risk Assessment: Theory, Methods, and Applications. Risk Assessment: Theory, Methods, and Applications. https://doi.org/10.1002/9781119377351.

Rogers, Everett M., Arvind Singhal, and Margaret M. Quinlan. 2019. "Diffusion of Innovations." In An Integrated Approach to Communication Theory and Research, Third Edition. https://doi.org/10.4324/9780203710753-35.

Sarran, Priya, Delia Clark, and Kathy Mendonca. 2011. "Change Management Toolkit: Tips, Tools, and Techniques for Leading a Successful Change Initiative." Berkley. H ttps://hr.berkeley.edu/sites/default/files/change_management_toolkit.pdf.

Shea, Christopher M., Sara R. Jacobs, Denise A. Esserman, Kerry Bruce, and Bryan J. Weiner. 2014. "Organizational Readiness for Implementing Change: A Psychometric Assessment of a New Measure." Implementation Science 9 (1). https://doi.org/10.1186/1748-5908-9-7.

Smith, Rachael, and Jayne Duffy. 2010. "Developing a Competent and Flexible Workforce Using the Calderdale Framework." International Journal of Therapy and Rehabilitation. https://doi.org/10.12968/ijtr.2010.17.5.47844.

Weiner, Bryan J. 2009. "A Theory of Organizational Readiness for Change." Implementation Science 4 (1). https://doi.org/10.1186/1748-5908-4-67.

World Health Organization. 2020. “Quality Health Services: A Planning Guide.” Geneva. https://www.who.int/publications/i/item/9789240011632.

Yankam, Brenda Mbouamba, Oluwafemi Adeagbo, Hubert Amu, Robert Kokou Dowou, Beryl Gillian Mbouamba Nyamen, Samuel Chinonso Ubechu, Pascal Georges Félix, Ngwayu Claude Nkfusai, Oluwaseun Badru, and Luchuo Engelbert Bain. 2023. “Task Shifting and Task Sharing in the Health Sector in Sub-Saharan Africa: Evidence, Success Indicators, Challenges, and Opportunities.” Pan African Medical Journal 46. https://doi.org/10.11604/pamj.2023.46.11.40984.

SECTION C

SHIFT-SHARE 2.0 Prompts Workbook

The following table provides question-based prompts or cues to use the SHIFT-SHARE 2.0 as a self-assessment tool or planning guide. Prompts can help reflect on current understanding and viewpoints regarding TS/S. Multiple stakeholders can collaboratively explore and note implementation challenges and solutions, coming up with an implementation plan for TS/S. Rows are colour-coded to match the corresponding illustrations in the framework diagram.

Element	Prompts
**Core Considerations**	
Clinical safety	• Are the tasks you plan to shift or share, clinically safe to do?<br>• Do you have clinical guidelines for staff in new roles?<br>• Do you have protocols in place to manage complications?
Patient-centredness	• Is your TS/S designed around patient needs?<br>• Do patients have input into how services are delivered?<br>• Have you considered how TS/S affect the patient experience?
Ethics	• Have you considered ethical implications of TS/S?<br>• Do you have informed consent about who provides care?<br>• Have you thought about equity and fairness?
Stakeholder feedback	• Are there channels for feedback?<br>• Do you act on the feedback received?<br>• Do you get back to the stakeholders, telling them how their input influenced decisions?
Workforce incentivisation	• Have you thought about meaningful ways to give staff incentives for taking on new roles via TS/S?<br>• Do you recognise and reward additional responsibilities?<br>• Have you thought about financial and non-financial motivators?
**Stage 01: Situational Analysis**	
**Needs Assessment**	
Patient needs	• Which services are most needed but least available or accessible by your community?<br>• Have you identified populations whose needs are underserved?<br>• Have you talked to patients about their unmet needs?
Care needs	• Do you know which services are delayed or unavailable?<br>• Do you know which aspects of care consume most provider time?<br>• Can you identify care gaps that TS/S could address?
System and process needs	• Have you mapped your current workflows and processes?<br>• What bottlenecks exist in service delivery?

	• Have you identified processes which could be shifted or shared?
Workforce requirements	• How many staff are needed to meet service demands?<br>• Have you identified skill gaps in your current workforce?<br>• Can you list the competencies needed for the key tasks?
Acute internal or external events	• How do emergency situations affect workforce capacity?<br>• Do you have plans for staff shortages during crises?<br>• Have you identified which tasks are critical during emergencies?
Compliance	• Do you know the relevant regulations for TS/S in your setting?<br>• Are there professional standards related to task redistribution?<br>• Have you considered any legal barriers to TS/S in your context?
**Readiness Assessment**	
Financial capacity	• Do you have budget for training on new tasks?<br>• Have you calculated costs of implementing TS/S?<br>• Can you sustain funding for training, support and supervision?
Infrastructural capacity	• Do you have physical spaces for training and new service delivery?<br>• Do you have the necessary equipment, or can you get it?<br>• Does your work context support the proposed workflow under TS/S?
Workforce capabilities	• Have you checked the current skill levels of your staff?<br>• Do you know who is interested in expanding their roles via TS/S?<br>• Is it possible that some staff could mentor others?
System constraints	• Have you identified organisational policies that might block TS/S?<br>• Are there any technological limitations which can affect implementation?<br>• Have you considered space or time constraints?
Commitment to change	• Have you gauged staff willingness to adopt new roles under TS/S?<br>• Do your leaders demonstrate support for TS/S?<br>• Have you identified potential champions for change?
Political will	• Have you thought about engaging with key decision-makers?<br>• Do local health authorities support your planned TS/S?<br>• Have you identified any political barriers to implementation?
Initial risk identification	• Have you brainstormed potential roadblocks or dead ends to TS/S?<br>• Which tasks carry the highest risk if shifted or shared?<br>• Have you thought about patient safety risks at this early stage?
**Stage 02: Preparation**	
**Task Analysis**	
Current state analysis	• Have you documented the current workflows or how services are currently delivered?<br>• What are the strengths and weaknesses in current approaches?<br>• Which tasks are suitable for shifting or sharing?

Workforce competency analysis	• Have you identified competency gaps that need addressing?<br>• What are the training needs of the staff?<br>• Have you documented these skillsets that are required for each task?
Continued risk identification	• Have you reviewed risks as your planning progresses?<br>• Do you discuss risks openly with team members?<br>• Have you identified new risks that were not apparent initially?
**Communication and Collaboration**	
Stakeholder engagement	• Have you identified all stakeholders affected by TS/S?<br>• Do you have methods to involve them in planning?<br>• Have you addressed concerns raised by different groups?
Needs communication	• Have you clearly explained why TS/S is necessary?<br>• Do others understand the benefits expected out of TS/S?<br>• Have you communicated how patients will benefit?
**Stage 03: Risk Signals**	
**Risk Management**	
Risk analysis	• Can you prioritise risks by type, severity or their likelihoods?<br>• What are the financial, worker safety, and legal risks to doing TS/S?<br>• Do you have systems to monitor these during TS/S's implementation?
Risk mitigation	• Are there strategies for tackling each major risk?<br>• Do you have backup plans if things go wrong?<br>• Have you created safety protocols for clinical risks?
**Stage 04: Capacity Building**	
**Training and Mentorship**	
Competency-focused training	• Does your planned training focus on required (practical-based) skills?<br>• Do you have qualified trainers available?<br>• Do you check competency-levels before allowing practice?
Continuous curriculum development	• Do you revisit training modules to update them based on feedback?<br>• How do you incorporate new evidence into your training?<br>• Do you adapt training to address identified gaps?
Active supervision and feedback	• Have you made regular supervision arrangements?<br>• Have you established mentorship pairs or groups?<br>• Do supervisors know how to give constructive feedback?
Accountability and responsibility	• Is it defined who is responsible for each task being shifted or shared?<br>• Do staff understand their scope of practice?<br>• Have you documented decision-making authorities?
**Building Support Systems**	
Governance and leadership	• What governance systems need to be in place for TS/S's success?<br>• Do you have clear leadership roles defined?<br>• Are there pathways for addressing implementation challenges?

Management and administration	• Have you developed administrative processes to support TS/S?<br>• Do you have systems for scheduling and reporting?<br>• How do you plan to make the documentation processes strong?
Technological support	• What technologies could support TS/S?<br>• Do staff have access to necessary equipment and systems?<br>• Have you provided training on these technologies?
Policy and regulatory support	• Are organisational policies updated to reflect new roles under TS/S?<br>• Do you advocate for supportive regulatory frameworks?<br>• Have you documented scope of practice guidelines?
**Stage 05: Monitoring and Evaluation**	
**Quality and Safety Checks**	
Coverage and access to care	• Has there been any improvement in service availability?<br>• Do you track how many patients receive shifted or shared services?<br>• Do underserved populations have better access now because of TS/S?
Healthcare quality	• Have you established quality indicators for shifted or shared tasks?<br>• Is there a possibility to compare outcomes before and after TS/S?<br>• Is there a plan to assess the impact of task shifting?
Patient satisfaction	• Have you created mechanisms to assess patient satisfaction?<br>• How often do you collect patient feedback?<br>• Do you address concerns raised by them?
Workforce satisfaction	• Have you surveyed staff about their experiences with new roles?<br>• Do you check for burnout or stress?<br>• Are there spaces or forums for staff to share concerns?
Cost effectiveness	• Have you calculated the costs and benefits of TS/S?<br>• Do you track resource utilisation?<br>• Have you compared financial outcomes to your projections?
Unintended consequences	• Have you identified any unexpected effects of TS/S?<br>• How do you detect unintended outcomes?<br>• Have you documented and reported positive and negative effects?
Reversibility	• Have there been instances of TS/S being reversed to care as before?<br>• Are there processes to modify or reverse TS/S on purpose if needed?<br>• Do you know which indicators would trigger these considerations?
**Stage 06: Maintenance and Diffusion**	
**Sustainability and Scalability**	
Reproducibility	• Have you documented your model of TS/S in detail for reproducing or duplicating it again in the same setting?<br>• Do you know which elements are essential versus adaptable?
Operational sustainability	• Have you integrated new processes into routine operations?<br>• Have you secured continued leadership support?<br>• What are your plans for maintaining TS/S over time?

Financial sustainability	• Have you identified funding for long-term viability of TS/S?<br>• Have you made the business case for continued investment?
Scalability	• Have you considered if which aspects of your approach to TS/S could work at a larger scale, perhaps in other similar contexts?<br>• Do you know what resources would be needed to expand?
**Knowledge sharing**	
Change in stakeholder perspectives	• Do you track how stakeholder views on TS/S have evolved?<br>• Have you leveraged positive changes in perspective to strengthen support for TS/S?
Transferability	• Have you documented your lessons for others to learn from?<br>• Have you created resources to help others implement similar TS/S?<br>• Do you know what contextual factors influence success?
